# Combination of the Topical Photodynamic Therapy of Chloroaluminum Phthalocyanine Liposomes with Fexinidazole Oral Self-Emulsifying System as a New Strategy for Cutaneous Leishmaniasis Treatment

**DOI:** 10.3390/pharmaceutics16040509

**Published:** 2024-04-07

**Authors:** Raphaela Ariany Silva, Danielle Soter Damasio, Larissa Dutra Coelho, Eliane de Morais-Teixeira, Celso M. Queiroz-Junior, Paulo Eduardo Souza, Ricardo Bentes Azevedo, Antônio Tedesco, Lucas Antônio Ferreira, Mônica Cristina Oliveira, Marta Gontijo Aguiar

**Affiliations:** 1 Department of Pharmaceutical Products, Faculty of Pharmacy, Federal University of Minas Gerais, Belo Horizonte 31270-901, Brazil; raphaelaassilva@gmail.com (R.A.S.); dansoter@gmail.com (D.S.D.); larissadc16@gmail.com (L.D.C.); lucaufmg@gmail.com (L.A.F.); 2 Clinical Research and Public Policy Group on Infectious and Parasitic Diseases, Instituto René Rachou, Fundação Oswaldo Cruz—FIOCRUZ, Belo Horizonte 330190-002, Brazil; eliane.teixeira@fiocruz.br; 3 Department of Morphology, Institute of Biological Sciences, Federal University of Minas Gerais, Belo Horizonte 31270-901, Brazil; cmqj@yahoo.com.br; 4 Laboratory of Software and Instrumentation in Applied Physics and Laboratory of Electron Paramagnetic Resonance, Institute of Physics, University of Brasília, Brasília 70910-900, Brazil; psouza@unb.br; 5 Nanobiotechnology Laboratory, Institute of Biological Sciences, University of Brasília, Brasília 70910-900, Brazil; razevedo@unb.br; 6 Department of Chemistry, Center of Nanotechnology and Tissue Engineering—Photobiology and Photomedicine Research Group, Faculty of Philosophy, Sciences and Letters of Ribeirão Preto, University of São Paulo, Ribeirão Preto 14040-900, Brazil; atedesco@usp.br

**Keywords:** chloroaluminum phthalocyanine, liposomes, fexinidazole, self-emulsifying drug release system, cutaneous leishmaniasis, combined therapy

## Abstract

Cutaneous leishmaniasis (CL) is a neglected tropical disease. The treatment is restricted to drugs, such as meglumine antimoniate and amphotericin B, that exhibit toxic effects, high cost, long-term treatment, and limited efficacy. The development of new alternative therapies, including the identification of effective drugs for the topical and oral treatment of CL, is of great interest. In this sense, a combination of topical photodynamic therapy (PDT) with chloroaluminum phthalocyanine liposomes (Lip-ClAlPc) and the oral administration of a self-emulsifying drug delivery system containing fexinidazole (SEDDS-FEX) emerges as a new strategy. The aim of the present study was to prepare, characterize, and evaluate the efficacy of combined therapy with Lip-ClAlPc and SEDDS-FEX in the experimental treatment of *Leishmania (Leishmania) major*. Lip-ClAlPc and SEDDS-FEX were prepared, and the antileishmanial efficacy study was conducted with the following groups: 1. Lip-ClAlPc (0.05 mL); 2. SEDDS-FEX (50 mg/kg/day); 3. Lip-ClAlPc (0.05 mL)+SEDDS-FEX (50 mg/kg/day) combination; 4. FEX suspension (50 mg/kg/day); and 5. control (untreated). BALB/c mice received 10 sessions of topical Lip-ClAlPc on alternate days and 20 consecutive days of SEDDS-FEX or FEX oral suspension. Therapeutical efficacy was evaluated via the parasite burden (limiting-dilution assay), lesion size (mm), healing of the lesion, and histological analyses. Lip-ClAlPc and SEDDS-FEX presented physicochemical characteristics that are compatible with the administration routes used in the treatments. Lip-ClAlPc+SEDDS-FEX led to a significant reduction in the parasitic burden in the lesion and spleen when compared to the control group (*p* < 0.05) and the complete healing of the lesion in 43% of animals. The Lip-ClAlPc+SEDDS-FEX combination may be promising for the treatment of CL caused by *L. major.*

## 1. Introduction

Leishmaniasis is considered a serious public health problem and is distributed worldwide. The main forms of the disease are cutaneous leishmaniasis (CL), visceral leishmaniasis, and mucocutaneous leishmaniasis. CL is endemic in about 87 countries around the world, and it is generally limited to an ulcer that self-heals over about a year; however, in some cases, it can cause scarring and disfigurement [1,2]. Geographically, CL can be divided into ‘Old World’ and ‘New World’ leishmaniasis. In Old World CL is predominantly caused by *Leishmania (Leishmania) major* [3].

The chemotherapeutic arsenal for CL consists of drugs, such as meglumine antimoniate (Sb^V^), pentamidine, amphotericin B, and miltefosine. These drugs have limitations such as low efficacy, the potential development of resistance, parenteral administration, and the need for hospitalization, making adherence difficult and increasing related costs. In addition, these treatments can cause serious side effects, such as hematological, pancreatic, and cardiac toxicity and teratogenicity [4]. The World Health Organization (WHO) recommends the investigation of alternative drugs and topical (local) and systemic (oral) formulations.

Fexinidazole (FEX) exhibited activity in vitro and in vivo, demonstrating its potential in the oral therapy of leishmaniasis [5,6,7]. However, FEX is sparingly soluble in water, which can be a limiting factor for its oral absorption. A self-emulsifying drug delivery system (SEDDS) is considered a potential carrier for oral administration, and it is a technology based on an isotropic mixture of lipids, surfactants, and co-surfactants, which forms a fine oil-in-water emulsion, in an aqueous medium after slight agitation. The SEDDS has gained interest in recent years for overcoming the challenges of the poor and irregular oral bioavailability of hydrophobic drugs [8,9,10]. This improvement can be explained by factors such as the following: no step being required for dissolution, greater interfacial surface area due to the fine dispersion of globules, and increased membrane fluidity [9,11,12].

Photodynamic therapy (PDT) is based on the photoactivation of photosensitizer molecules (PSs), which can generate cytotoxic reactive oxygen species (ROS), such as singlet oxygen (^1^O_2_) and free radicals when irradiated by visible light at a specific wavelength. The species can damage cellular organelles causing cell death and tissue destruction irreversibly [13]. Chloroaluminum phthalocyanine (ClAlPc) is a great PS due to its chemical stability, strong absorption spectrum in the red-light range (670–780 nm), high efficiency in generating ROS, and excellent activity against different Leishmania species after PDT [13,14,15]. The performance of a PS as an antileishmanial drug for topical application can be improved if an appropriate carrier is used for transdermal delivery. Liposomes are a vesicular nanosystem, constructed by the self-assembly of amphipathic molecules in a bilayer in which the hydrophilic head groups face the outer aqueous environment and the hydrocarbon chains assemble within the hydrophobic interior. The incorporation of hydrophilic and lipophilic drugs into liposomes makes them suitable nanocarriers for drug delivery through different administration routes [16]. Liposomes are used as carriers of some antileishmanial drugs, such as AmB, Sb^V^, and miltefosine. The incorporation of ClAlPc into liposomes can facilitate its access to Leishmania-infected cells, increase its photoactivity by conserving its monomeric structure, and reduce toxicity [17].

Thus, this work reports the evaluation of the therapeutical efficacy of chloroaluminum phthalocyanine-loaded liposomes (Lip-ClAlPc) combined with the oral treatment of FEX-loaded SEDDS (SEDDS-FEX) as a new therapy for the treatment of CL caused by *L. (L.) major*.

## 2. Materials and Methods

### 2.1. Preparation of Formulations

Lip-ClAlPcs composed of egg phosphatidylcholine (Lipoid GmbH, Ludwigshafen, Germany) were obtained as previously published by Lopes and collaborators [15], with the following modifications: after preparation, the vesicles were submitted to extrusion using polycarbonate membranes (0.4 μm, 10 times) and purified by ultracentrifugation at 150,000× *g* at 4 °C for 190 min (Optima^®^ L-80XP; Beckman Coulter, Indianapolis, IN, USA). Finally, the obtained pellet was resuspended to a final volume three times lower than the original.

To prepare SEDDS-FEX, surfactants and co-surfactants were weighed (Tween 80^®^, Span 80^®^, and Kolliphor RH 40^®^) (Sigma Chemical Company, Saint Louis, MO, USA) and homogenized for 2 min on a magnetic stirrer. Then, FEX (Centipharm, Grasse, France) (10 mg/g) was added, and the mixture was stirred for 2 min. After homogenization, the medium-chain triglyceride (MTC) (Lipoid GmbH, Ludwigshafen, Germany) was added slowly and stirred for another 2 min. As the last step of the process, the formulation was placed in an ultrasound bath for 30 min at 40 °C to obtain an isotropic mixture and then kept under magnetic stirring for 24 ± 2 h at room temperature [7]. The selection of the components used in the preparation of SEDDS-FEX was carried out considering the toxicity, solvent capacity, miscibility, physical state at room temperature, and stability. 

FEX was weighed in a centrifuge tube (15 mL) containing glass spheres, along with Tween 80^®^, to improve the homogenization process during the preparation of the FEX suspension. Then, this mixture was homogenized in a vortex until the FEX was completely covered in the Tween 80^®^. Purified water was added, and the mixture was homogenized again by vortex until a suspension was obtained. The formulation was protected from light and stored at 4 °C for 7 days [6].

### 2.2. Physicochemical and Chemical Characterization of Formulations

The average diameter and polydispersity index (Pdi) of the systems were investigated via photon correlation spectroscopy, and the zeta potential (ZP) was determined via dynamic light scattering associated with electrophoretic mobility [7,15]. The measurements were performed using Zetasizer Nano ZS90 equipment (Malvern Instruments Ltd., Worcestershire, UK). The Lip-ClAlPc and SEDDS-FEX samples were diluted with a sodium chloride 0.9% (*w*/*v*) solution and Milli-Q water, respectively [7,15]. Measurements are performed in triplicate, and values are represented as mean ± standard deviation.

The identification of FEX crystals that are insoluble in SEDDS-FEX and FEX suspension samples was carried out using polarized light microscopy analysis and Microscope Optical Zeiss^®^ (San Francisco, CA, USA). The analyses were carried out at 100× magnification.

The encapsulation percentage (EP) of chloroaluminum phthalocyanine (ClAlPc) in liposomes was determined via the spectrophotometric method with detection at 674 nm (UV mini-1240, Shimadzu, Kyoto, Japan). Initially, the vesicles were opened with ethyl alcohol (95%) at a volume of 1 mL Lip-ClAlPc:2.3 mL ethyl alcohol 95%. The preparations were diluted in ethyl alcohol (95%). The concentration of ClAlPc in the liposome was determined using a regression equation obtained from the ClAlPc standard curve y = 0.467x − 0.0191 [15].

The EP of the ClAlPc was calculated according to the following equation:

$$\mathrm{EP}=\frac{\;[\mathrm{amount}\;\mathrm{of}\;\mathrm{ClAIPc}\;\mathrm{in}\;\mathrm{purified}\;\mathrm{liposomes}\;]}{\left[\mathrm{amount}\;\mathrm{of}\;\mathrm{CIAIPc}\;\mathrm{in}\;\mathrm{the}\;\mathrm{non-purified}\;\mathrm{liposomes}\right]}\times 100.$$

The FEX content in SEDDS was measured via the reversed-phase high-performance liquid chromatography (HPLC) method, as previously published by Damasio and collaborators [7]. The concentration of FEX in SEDDS was determined via the regression equation y = 34965x − 1093 obtained from the FEX standard curve [7].

### 2.3. In Vivo Antileishmanial Efficacy

For the in vitro propagation of *L. (L.) major* (MHOM/IL/80/Friendlin) promastigotes, complete Schneider’s medium (Merck, Darmstadt, Germany) was used, enriched with 20% fetal bovine serum (Gibco, Eggenstein, Germany). The promastigotes were maintained in culture until they reached the stationary phase of growth.

BALB/c female mice (age: 7 weeks) were shaved and inoculated subcutaneously with 0.02 mL of a dispersion containing 1 × 10^7^ promastigotes of *L. (L.) major* at the base of the tail [18]. The study was approved by the Ethics Committee for Animal Experimentation of the Universidade Federal de Minas Gerais (CEUA/UFMG—18/2020).

After the development of homogeneous lesions (average diameter of about 8 mm), the animals were divided into 5 groups of treatment containing 7 animals per group: group 1—Lip-ClAlPc (0.05 mL); group 2—FEX suspension (50 mg/kg/day); group 3—SEDDS-FEX (50 mg/kg/day); group 4—Lip-ClAlPc (0.05 mL)+ SEDDS-FEX (50 mg/kg/day) combined; group 5—infected untreated animals (control).

The Lip-ClAlPc formulation was applied topically (10 doses) in the lesion and protected from light. After 15 min, the lesion was exposed to visible light irradiation for 20 min at a wavelength of 660 nm, releasing 0–95 J/cm^2^ at an intensity of 81 mW/cm^2^. The distance between the light source and the lesion was 5 cm. After the session, the residues of the formulation were removed using 0.9% (*w*/*v*) sterile saline [15]. The FEX suspension and SEDDS-FEX were administered daily (0.2 mL) for 20 consecutive days by oral gavage, with SEDDS-FEX being previously diluted in water (1:1) before administration [7]. For the control group, the animals did not receive treatment.

Lesion size was measured weekly using a digital caliper (Mitutoyo, São Paulo, Brazil). The size of the lesion was determined by the average value obtained between the longest line that could be traced from one border of the lesion to another and the line that bisected this distance at a 90° angle [18]. The lesions were photographed at the beginning and end of the treatments.

The body weight of animals was evaluated at the beginning of the treatment (0), day 7, day 14, and end of the treatment (21 days). Other signs such as piloerection were used as indicators of the toxicity of the treatment. Additional evaluations included checking the appearance of nodules and metastasis in other locations on the animal skin as signs of disease progression.

The parasite burden in the lesions, as well as in the spleen, was evaluated via the limiting dilution assay, as described by Aguiar and collaborators [19]. The results were expressed as the mean obtained from the triplicate.

After the euthanasia procedure, the skin samples of the lesions of mice were collected and fixed in 10% neutral buffered formalin (pH 7.2) for a minimum period of 48 h. After processing, the fragments of the lesions were embedded in paraffin, submitted to microtomy with 5 μm, and stained with hematoxylin and eosin (H&E). The images were obtained using a microcamera (Q-Color5, Olympus, Tokyo, Japan) coupled to a BX53 microscope (Olympus), and the Q-Capture Pro 7.0 software was used [20]. The inflammatory reaction was evaluated using a semiquantitative procedure assessing the presence of leukocytes in the dermis and hypodermis [21,22]. The inflammatory infiltrate score system was adapted as follows: 0 = absent, no mononuclear cell exudate (apparently normal dermis); 1 = slight, diffuse mononuclear exudate in the upper dermis (1–9 cells per field/20 fields); 2 = moderate, a diffuse or focal mononuclear exudate around the vessels, glands, and hair follicles in the deep dermis or hypodermis (10–30 cells per field/20 fields); 3 = intense, a severe diffuse or focal mononuclear exudate around the vessels, glands, and hair follicles in deep dermis or hypodermis; 4 = intense, severe, and widespread inflammatory infiltrate in all dermis (>30 cells per field/20 fields). The analyzed morphological parameters of the epidermis were acanthosis, dyskeratosis, papillomatosis, and exocytosis: 0 = absent; 1 = present [23]. The total score was calculated by the sum of all parameters evaluated in the epidermis, dermis, and hypodermis. Histopathological analysis was performed using a single-blinded model [22].

**Figure 1:**
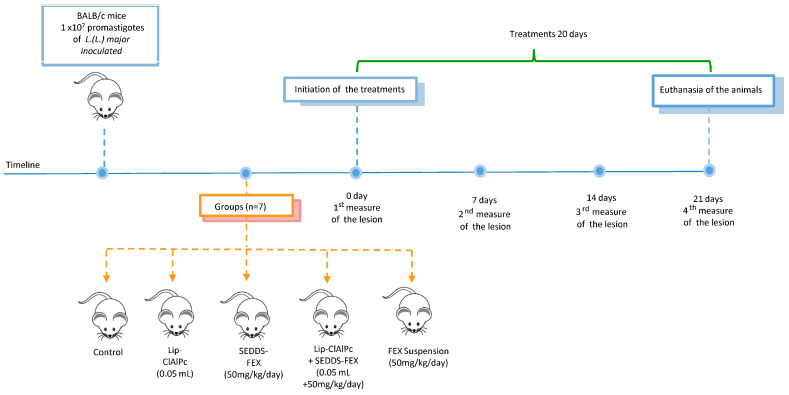
Timeline course and treatment regimen. Female BALB/c mice were infected with *L. (L.) major* promastigotes and received 10 sessions of topical Lip-ClAlPc on alternate days and 20 consecutive days of SEDDS-FEX or FEX suspension oral in isolated or combined treatments. Therapeutical efficacy was evaluated by measuring the lesion size (0, 7, 14, and 21 days), the parasite burden, and histological analysis after euthanasia.

The course of the timeline and treatment regimen is schematized in Figure 1.

### 2.4. Statistical Analysis

The data were processed using GraphPad Prism software. Normality and homogeneity of variance were assessed using the Kolmogorov–Smirnov and Bartlett’s tests, respectively. Then, the comparison of parasite loads, weights of animals, and lesion sizes among the groups was evaluated via ANOVA, and in the case of statistical differences, the results were confirmed via Tukey’s test. For histopathological analysis, a comparison among groups was performed using the Kruskal–Wallis test. The difference was considered significant when the *p*-value was <0.05.

## 3. Results

### 3.1. Formulations Characterization

The diameter, polydispersion index, zeta potential, and concentration measured for the two formulations (Lip-ClAlPc and SEDDS-FEX) prepared for the in vivo study are shown in Table 1.

Lip-ClAlPc and SEDDS-FEX formulations presented suitable characteristics for topical and oral administration, respectively.

**Figure 2:**
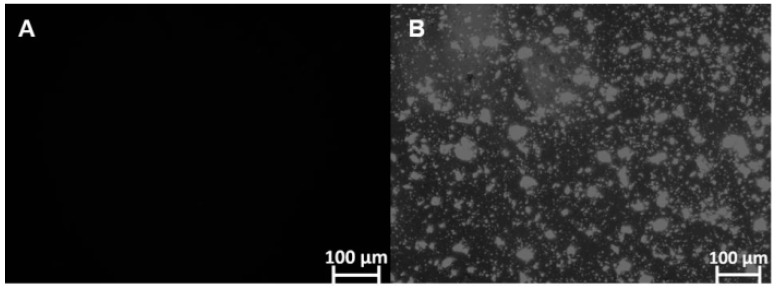
The identification of FEX crystals that are insoluble in SEDDS-FEX and FEX suspensions (undiluted samples) was carried out using polarized light microscopy analysis and the Optical Zeiss^®^ microscope. The analyses were carried out at 100× magnification: (**A**) SEDDS-Fex and (**B**) Fex suspension.

Furthermore, microscopic analysis showed that FEX is soluble in SEDDS-FEX (Figure 2A), while in the suspension, it is possible to verify the presence of FEX crystals (Figure 2B). FEX has low solubility in water, which may be a limiting factor for its absorption when administered orally. In fact, SEDDS-FEX was able to increase the solubility of FEX by keeping it in the dissolved state as a colloidal dispersion.

### 3.2. In Vivo Antileishmanial Efficacy

**Figure 3:**
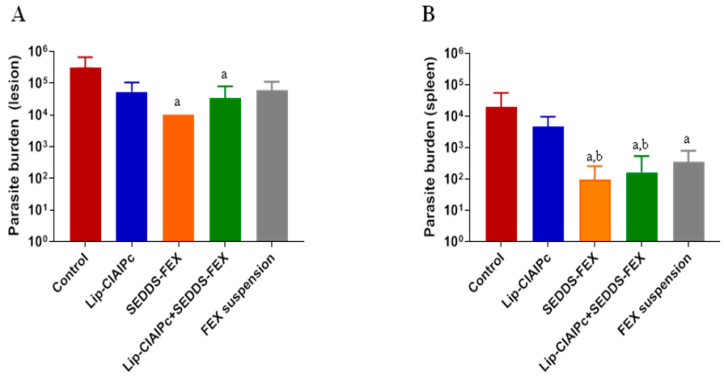
In vivo efficacy of different treatments in *L. (L.) major*. Female BALB/c mice were infected with *L (L.) major* pomastigotes in the base of the tail. The treatments used were Lip-ClAlPc, SEDDS-FEX, Lip-ClAlPc+SEDDS-FEX, FEX suspension, and control group (untreated). The Lip-ClAlPc and Lip-ClAlPc+SEDDS-FEX groups received 10 sessions of topical Lip-ClAlPc on alternate days and 20 consecutive days of SEDDS-FEX or FEX oral suspension in isolated or combined treatments. One day after the end of the treatments, the parasite burden was determined via the limiting dilution method. (**A**) Parasite burden in lesions. The letter “a” indicates a statistically significant difference in relation to the control group (*p* < 0.05). (**B**) Parasite burden in the spleen. The letters “a” and “b” indicate statistically significant differences in relation to the control and Lip-ClAlPc groups, respectively (*p* < 0.05). The bars represent the averages and standard deviations (*n* = 7).

Initially, the animals were experimentally infected with *L. (L.) major* and the development of lesions followed the prognosis described in the literature [18,24]. It is important to highlight that when evaluating the parasite burden in the lesion, only the SEDDS-FEX (1 × 10^4^), and Lip-ClAlPc+SEDDS-FEX (3 × 10^4^) groups showed a significant reduction (*p* < 0.05) in relation to the control group (3 × 10^5^) (Figure 3A). However, when comparing all treatments, there is no significant difference between them (*p* > 0.05) (Figure 3A). The groups treated with the Lip-ClAlPc and FEX suspensions exhibited parasite burdens of 5 × 10^4^ and 6 × 10^4^, respectively.

The parasite burden in the spleen is presented in Figure 3B. In fact, all formulations containing fexinidazole that were administered at doses of 50 mg/kg/day (Lip-ClAlPc+SEDDS-FEX (6 × 10^2^), SEDDS-FEX (1 × 10^2^), and FEX suspensions (8 × 10^2^)) were able to significantly reduce the parasite load in the spleen (Figure 3B) when compared with the control group (2 × 10^4^) (*p* < 0.05). It was also possible to observe a statistical difference in the Lip-ClAlPc group (6 × 10^3^) when compared with the SEDDS-FEX and Lip-ClAlPc+SEDDS-FEX groups (*p* < 0.05) (Figure 3B). However, the Lip-ClAlPc group does not differ statistically from the FEX suspension group (*p* > 0.05). It is interesting to note that the SEDDS-FEX treatment led to a greater reduction in parasite loads in the lesion and the spleen than the FEX suspension treatment. This finding is probably related to increased bioavailability, which may be related to the complete solubilization of FEX when administered in the form of SEDDS-FEX.

**Figure 4:**
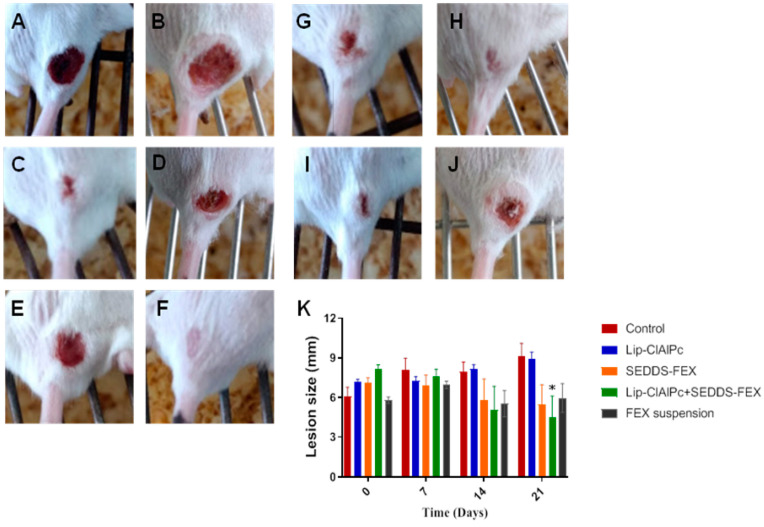
In vivo efficacy of different treatments in *L. (L.)* *major*. Female BALB/c mice were infected with *L (L.) major* pomastigotes in the base of the tail. The treatments used were Lip-ClAlPc, SEDDS-FEX, Lip-ClAlPc+SEDDS-FEX, FEX suspension, and control group (untreated). The Lip-ClAlPc and Lip-ClAlPc+SEDDS-FEX groups received 10 sessions of topical Lip-ClAlPc on alternate days and 20 consecutive days of SEDDS-FEX or FEX suspension in isolated or combined treatments. Images of the macroscopic aspect of the lesions (**A**–**J**). Control group: infected and untreated animals at 0 days (**A**) and 21 days (**B**). Animal treated with Lip-ClAlPc at 0 days (**C**) and 21 days (**D**). Animal treated with SEDDS-FEX at 0 days (**E**) and 21 days (**F**). Animal treated with Lip-ClAlPc+SEDDS-FEX at 0 days (**G**) and 21 days (**H**). Animal treated with the FEX suspension at 0 days (**I**) and 21 days (**J**). (**K**) Monitoring of average lesion size in response to different treatments. Lesion size is shown as the average and standard error of the mean. * Indicates statistically significant difference in relation to the control group (*p* < 0.05) on the 21st day (*n* = 7).

At the beginning of the treatment, the animals presented lesions with an average diameter equal to the following: control group (6.1 ± 1.9 mm), Lip-ClAlPc (7.2 ± 0.6 mm), SEDDS-FEX (7.1 ± 1.0 mm), Lip-ClAlPc+SEDDS-FEX (8.1 ± 0.9 mm), and FEX suspension (5.8 ± 0.7 mm); there were no significant differences between all groups (*p* > 0.05) (Figure 4K). It is possible to verify that the Lip-ClAlPc+SEDDS-FEX group was the only one that significantly reduced the size of the lesion in relation to the control group on the 21st day (*p* < 0.05) (Figure 4K). The images of the animals lesions at the beginning of treatment (day 0) and at the end (21 days) are presented in Figure 4: control group (Figure 4A,B), Lip-ClAlPc (Figure 4C,D), SEDDS-FEX (Figure 4E,F), Lip-ClAlPc+SEDDS-FEX (Figure 4G,H), and FEX suspension (Figure 4I,J).

**Figure 5:**
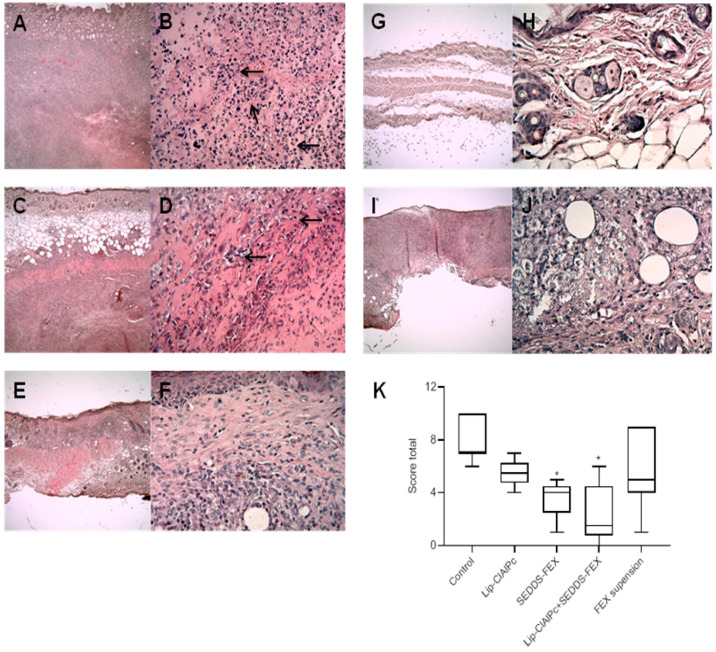
Histopathological analysis (H&E) was performed in the control group (**A**,**B**), Lip-ClAlPc (**C**,**D**), SEDDS-FEX (**E**,**F**), Lip-ClAlPc+SEDDS-FEX (**G**,**H**), and FEX suspension (**I**,**J**). The first image on the right presents 4× magnification, and the second image on the left the image presents 40× magnification. Arrows indicate the regions with inflammatory processes. (**K**) Total score of the histopathological analysis. Data presented as median, maximum, and minimum. The groups treated with SEDDS-FEX and Lip-ClAlPc+SEDDS-FEX exhibited statistical differences when compared to the control group (* *p* < 0.05) (*n* = 7).

The histopathological analysis of skin lesions of the mice in the control group revealed extensive inflammatory and necrotic lesions (Figure 5A,B). The lesion was characterized by large areas of necrosis and ulceration, as well as a mixed inflammatory infiltrate, with a predominance of mononuclear macrophages in deeper regions of the dermis. Vacuolated and parasitized macrophages were also detected, as well as the points of dystrophic calcification. In the group that received Lip-ClAlPc treatment (Figure 5C,D), the changes detected were less intense, and no changes were observed in the epidermis, including the absence of ulcerated areas in almost all samples. The inflammatory infiltrate and areas of necrosis ranged from moderate. Similar characteristics were seen in the group that received the treatment with SEDDS-FEX (Figure 5E,F).

The treatment with Lip-ClAlPc+SEDDS-FEX (Figure 5G,H) attenuated the lesion phenotype with no epidermal lesions, and the absence of necrosis was observed in most samples. Finally, in the FEX suspension-treated group (Figure 5I,J), a variation in the lesion was observed among the animals, with some presenting intense alterations as described in the control group while others presented mild lesions in limited areas. The total score is shown in Figure 5K, where we can see that the SEDDS-FEX and Lip-ClAlPc+SEDDS-FEX-treated groups exhibited a significant difference when compared to the control group (*p* < 0.05).

**Figure 6:**
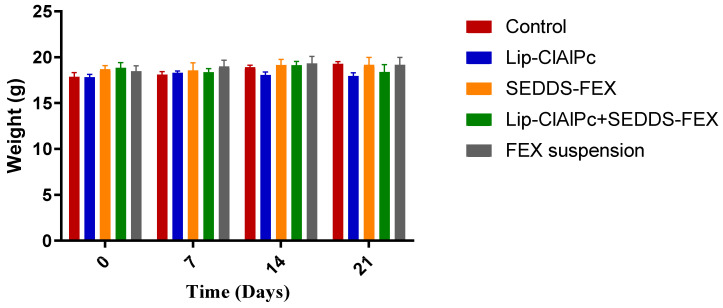
Evaluation of the weight of BALB/c mice infected with *L. (L.) major* and submitted to different treatments. Female BALB/c mice were infected with *L (L.) major* promastigotes at the base of the tail. After the development of ulcerated lesions, the animals were treated with the Lip-ClAlPc, SEDDS-FEX, Lip-ClAlPc+SEDDS-FEX, FEX suspension, and Control (untreated). Animals were weighed at baseline (0 days) and on days 7, 14, and 21 after starting treatment. The bars represent means and standard deviations. Comparisons between the weights at all times were not statistically significant (*p* > 0.05).

The comparisons of the average body weight of the animals of each group on days 0, 7, 14, and 21 were not statistically different (*p* > 0.05) (Figure 6). Other signs of toxicity evaluated, such as piloerection and death, were not observed.

## 4. Discussion

*L. (L.) major* is one of the main species causing CL in the Mediterranean basin, Middle East, and Africa. Co-infection with HIV has led to atypical manifestations of CL, as was observed in an outbreak related to this species in Burkina Faso [2]. Therefore, these factors show the importance of searching for new therapeutic alternatives for the treatment of CL caused by this species. It is important to highlight that each species of *Leishmania* presents specific biochemical and molecular characteristics, and their sensitivity to drugs is different [25]. Published studies reported the in vivo efficacy of treatments with FEX and ClAlPc against different species that cause leishmaniasis, such as *L. donovani*, *L. infantum*, *L. amazonensis,* and *L. braziliensis*, showing promising results [5,6,7,13,15,26]. However, until now, no study has been found that evaluated the effectiveness of these treatments against *L. (L.) major*. On another point, combination therapy is a very promising alternative to improve the efficacy of the treatments against leishmaniasis. Through this strategy, it is possible to test drugs with synergistic or additive activity, which can reduce the duration of therapy or decrease the effective doses, allowing treatment with less toxicity than therapies with isolated drugs [27,28].

The physicochemical and chemical properties of nanostructures, such as size and composition, are factors that can influence their interaction with the organism, potentially impacting the stability, effectiveness, and toxicity of these systems [29,30]. When evaluating the size obtained for the Lip-ClAlPc (348 nm), the size obtained is compatible with the opening of the membrane (0.4 µM) used for their extrusion. It is important to consider that liposomes with sizes greater than 500 nm may compromise their use as a drug-release vehicle [26]. SEDDS-FEX, after dilution, showed globules with an average size of around 100 nm (Table 1), which indicates a compact arrangement of the surfactant at the oil–water interface [31], a relevant parameter in improving drug absorption after oral administrations, as discussed by Chatterjee and coworkers (2016) [8]. The Pdi value provides information about the homogeneity of the size distribution of nanosystems. The values found for Lip-ClAlPc and SEDDS-FEX formulations were at most equal to 0.3, which indicates the obtainment of monodisperse systems, further contributing to an adequate therapeutic response, and these systems are appropriate for topical and oral administration [26]. High values of ZP induce electrostatic repulsion, preventing coalescence and improving stability [29]. The ZP values obtained were slightly negative. It is important to highlight that the Lip-ClAlPc and SEDDS-FEX formulations were stable for 2 months at 4 °C and 6 months at 25 °C, respectively [7,15].

As observed through microscopic analysis, no FEX crystals were present in the SEDDS-FEX formulation. This finding is desirable to ensure that the emulsion formed after diluting the SEDDS does not present the precipitation of the active pharmaceutical ingredient, as the presence of insoluble crystals in the medium can lead to a delay or decrease in its absorption [32,33]. Damasio and collaborators (2023) [7] also observed that for this formulation, at dilutions of 1:200 in water and simulated gastric or intestinal fluids, FEX remained soluble.

Skin lesions are easily accessible for light sources and provide an opportunity for the introduction of PDT, which is interesting for the topical treatment of CL [34]. In this study, an improvement was observed in all parameters evaluated after the Lip-ClAlPc+SEDDS-FEX treatment compared with the control group. In vivo studies are an effective method for screening new treatments for leishmaniasis. It is important to note that BALB/c mice infected with *L. major* have been widely used for decades to test the efficacy of potential anti-leishmanial drugs, and they are considered a rigorous non-cure model in which only the most active drugs are effective. Thus, any improvement in the disease can be attributed to the effects of chemotherapy. The lesion cure model in BALB/c mice is highly reproducible and consistent, and it has some clinical similarities with human CL [35]. However, this criterion is not always correlated with a reduction in parasite burden, since part of the lesion is composed of inflammatory cells with amastigotes restricted to the layer dermal [36]. Interestingly, our results showed that treatment with the Lip-ClAlPc+SEDDS-FEX was able to reduce lesions, as well as parasite burden.

It is also interesting to note that the SEDDS-FEX treatment was able to lead to a greater reduction in the parasite load in the lesion than the FEX suspension treatment. This is probably because FEX is completely solubilized when administered in the form of SEDDS-FEX. According to the Biopharmaceutical Classification System, FEX is a class II drug and has low solubility and high permeability [37]; therefore, its low solubility is the limiting factor for absorption. When SEDDS-FEX is released into the lumen of the intestine, it can disperse to form a nanometer-sized emulsion, thereby improving the dissolution rate of the drug. Tarral and collaborators (2014) [38] showed that the bioavailability of FEX administered in tablet form in humans is approximately 25% lower than when administered in suspension form. The reduced absorption of FEX in tablet form is explained by the time required for disintegration and dissolution, as the rate of dissolution of the drug is the determining factor for the degree of absorption within a given period of gastric transit.

In the present study, the combined treatment of Lip-ClAlPc+SEDDS-FEX was able to significantly reduce the parasite burden in the lesion and spleen, in addition to the complete healing of the lesions in 43% of the animals. On the other hand, in previous studies conducted on species causing CL in the New World, a high dose was required to reduce the parasite burden in the organs evaluated, as well as for the healing of the lesions [6]. Despite the difference between the species, our results show a clinical improvement correlated with anti-leishmanial activity, which can be attributed to the combined effects of the drugs, as well as the form of drug delivery. Conventional drug delivery systems are not capable of targeted delivery, while solutions based on nanotechnology are the innovative approach to therapeutic advancement in the fight against leishmaniasis [11]. The industrial preparation of SEDDS is economical and simple, as it is almost like preparing the solution, using basic production equipment [10], while the proposed method for preparing liposomes was the most common (hydration of the lipid film) [16].

Drug discovery and development are expensive, time-consuming, and risky endeavors. Pharmaceutical companies show low interest in the development of anti-leishmanial drugs, most likely due to low financial returns. Therefore, the development of new formulations has become an interesting strategy for infectious and neglected diseases, especially in emerging countries [39,40].

## 5. Conclusions

In the present study, we observed both a reduction in parasitic burden in the regions evaluated (lesion and spleen) and a clinical improvement in lesions, with a reduction in size and healing when the animals were treated with the combination of Lip-ClAlPc+SEDDS-FEX. This finding indicates that this combination may be promising for the treatment of CL caused by *L. (L.) major.*

## Figures and Tables

**Table 1:** Physicochemical characteristics of Lip-ClAlPc and SEDDS-FEX formulations.

Formulations	Average Diameter (nm)	Polydispersity Index (Pdi)	Zeta Potential (mV)	EP (%) *	Concentration (mg/mL) **
Lip-ClAlPc	348 ± 14.6	0.3 ± 0.1	−2.5 ± 0.2	96.04 ± 0.31	-
SEDDS-FEX	66.5 ± 0.6	0.2 ± 0.01	−21.6 ± 0.3	-	9.17 ± 0.05

Data are expressed by the mean (*n* = 3) ± standard deviation. * Encapsulation percentage (EP) of ClAlPc in liposome. ** Concentration of FEX in SEDDS.

## Data Availability

Data are available in a publicly accessible repository that does not issue DOIs. Publicly available datasets were analyzed in this study. These data can be found here: http://hdl.handle.net/1843/41158, accessed on 4 April 2024.
